# Cost-effectiveness analysis of bevacizumab combined with lomustine in the treatment of progressive glioblastoma using a Markov model simulation analysis

**DOI:** 10.3389/fpubh.2024.1410355

**Published:** 2024-05-30

**Authors:** Zhaoyan Chen, Fangyuan Tian, Ying Zhang

**Affiliations:** Department of Pharmacy, National Clinical Research Center for Geriatrics, West China Hospital, Sichuan University, Chengdu, Sichuan, China

**Keywords:** progressive glioblastoma (GBM), bevacizumab, lomustine, first-line treatment, cost effectiveness

## Abstract

**Background:**

Progressive glioblastoma (GBM) is a malignancy with extremely poor prognosis. Chemotherapy is one of the approved systemic treatment modalities. The aim of this study is to assess the cost-effectiveness of using bevacizumab (BEV) in combination with lomustine (LOM) regimen for the treatment of progressive glioblastoma in China.

**Methods:**

The estimation results are derived from a multicenter randomized phase III trial, which demonstrated improved survival in GBM patients receiving BEV+LOM combination therapy. To calculate the incremental cost-effectiveness ratio (ICER) from the perspective of Chinese society, a Markov model was established. Univariate deterministic analysis and probabilistic sensitivity analysis were employed to address the uncertainties within the model.

**Results:**

Compared to LOM monotherapy, the total treatment cost for BEV+LOM combination therapy increased from $2,646.70 to $23,650.98. The health-adjusted life years (QALYs) for BEV+LOM combination therapy increased from 0.26 QALYs to 0.51 QALYs, representing an increment of 0.25 QALYs. The incremental cost-effectiveness ratio (ICER) was $84,071.12. The cost-effectiveness curve indicates that within the willingness-to-pay (WTP) range of $35,906 per QALY, BEV+LOM combination therapy is not a cost-effective treatment option for unresectable malignant pleural mesothelioma patients.

**Conclusions:**

Taken as a whole, the findings of this study suggest that, from the perspective of payers in China, BEV+LOM combination therapy as a first-line treatment for GBM is not a cost-effective option. However, considering the survival advantages this regimen may offer for this rare disease, it may still be one of the clinical treatment options for this patient population.

## Introduction

Progressive glioblastoma multiforme (GBM) refers to a particularly invasive and rapidly growing brain tumor, typically diagnosed at an advanced stage, exhibiting marked malignant features. This type of tumor often swiftly infiltrates surrounding brain tissues during its growth process, potentially metastasizing to other sites, thereby escalating the complexity, and difficulty of treatment. Clinically, progressive GBM typically manifests severe neurological symptoms in patients, such as headaches, nausea, and motor disturbances. Radiologically, these tumors typically present as large, irregular lesions with indistinct borders, potentially intermingling with adjacent normal brain tissues, posing significant challenges for treatment. Despite the partial efficacy of existing chemotherapy regimens in controlling the progression of progressive GBM, the highly invasive and malignant nature of this tumor renders its treatment inherently challenging, with generally poor prognoses for patients (1–4). A clinical study found that the addition of bevacizumab, while extending progression-free survival to some extent, did not confer a survival advantage over using temozolomide alone in patients with progressive GBM (5).

Bevacizumab is a monoclonal antibody that targets vascular endothelial growth factor (VEGF) in the bloodstream. It has been approved for the treatment of recurrent high-grade gliomas in the United States but has not been approved in Europe. For patients with recurrent high-grade gliomas, bevacizumab as monotherapy or in combination with chemotherapy drugs (such as temozolomide) has achieved imaging response rates of 30% to 40% (6–8). Most studies have also shown that the drug has corticosteroid-sparing effects in many patients, which can positively impact the quality of life for patients.

Currently, the global burden of medication remains significant, especially among the older adult population (9). Despite the significant improvement in progression-free survival and good tolerance seen with bevacizumab and lomustine in patients with progressive glioblastoma, the cost is high. In China, although bevacizumab has undergone national medical insurance negotiations, resulting in a reduction in price, the unit price of a standard 100 mg dose still remains high at $209. The completely self-funded treatment approach may lead patients to forgo or delay treatment, lower their quality of life, and put them at risk of bankruptcy. From a societal perspective, China is a developing country with insufficient medical facilities and relatively uneven regional economic development, particularly in the western regions. This disparity became even more pronounced following the outbreak of the COVID-19 pandemic in 2019 (10, 11). However, the advantages of this new treatment approach for GBM patients need to be considered. Therefore, this study intends to use a Markov model to evaluate the economics of this treatment regimen, providing necessary reference and data support to physicians, patients, and decision-makers.

## Materials and methods

### Target population

This study included patients whose baseline characteristics were consistent with a large-scale clinical trial (5). The strength of the methodology is its quick results and it does not require actual patients with all the ethical clearance procedures. The inclusion criteria were as follows: Patients who, at least 3 months after the completion of radiotherapy, were histologically confirmed to have glioblastoma multiforme with clear evidence of their first disease progression. Patients who had previously received anti-angiogenic therapy were excluded. Patients were randomly allocated in a 2:1 ratio to receive either combination therapy (bevacizumab at 10 mg/kg q2w, ivgtt, lomustine at 90 mg/m^2^ q6w) or monotherapy with lomustine (110 mg/m^2^ q6w, with a maximum dose of 200mg). Each cycle was defined as 6 weeks, with the 1^st^ day of medication considered Day 1 of each cycle.

### Model structure

**Figure 1:**
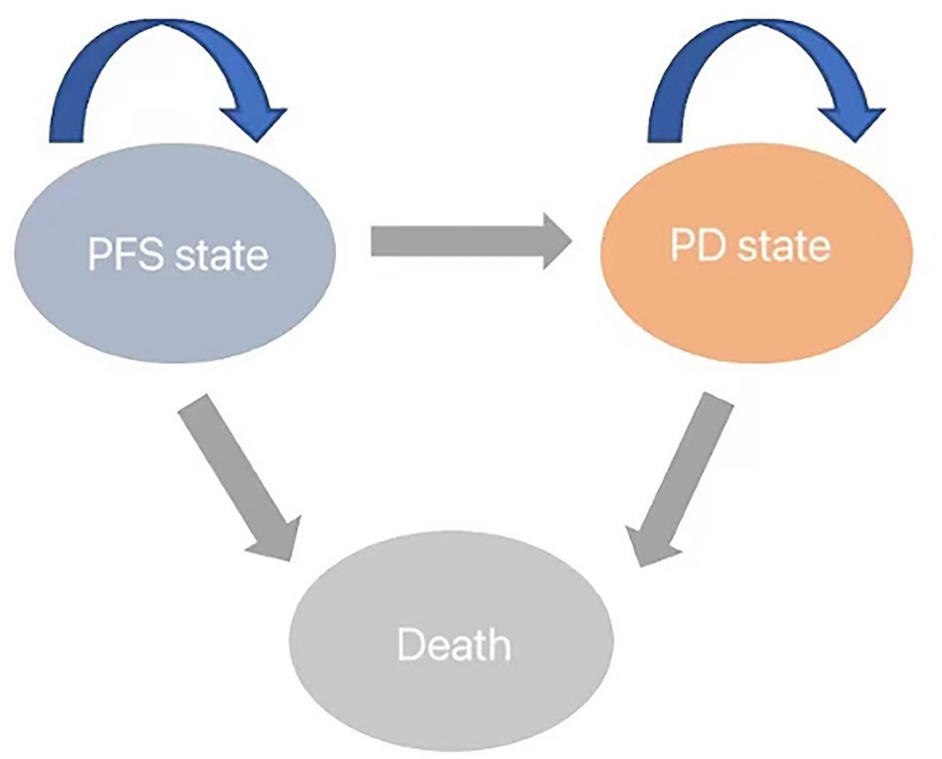
Diagram of transitions between health states.

We developed a Markov model to simulate the disease progression of patients with progressive glioblastoma under two treatment strategies. Initially, all unresected glioblastoma patients are in a progression-free survival (PFS) state, but over time, they transition to the next state or remain unchanged (Figure 1). The model assumes that patients in the progressive disease (PD) state cannot revert to the PFS state. At the conclusion of the simulation, all patients transition to the deceased state. The cycle length is set to 1 month based on clinical symptoms and disease progression. Monthly transition probabilities are derived from median survival estimates (Table 1) using the formula: P (1 month) = 1 – (0.5)^∧^(1/median time to event), which can be easily derived from the formulas P = 1 – e^∧^(-R) and R = –ln [0.5]/(time to event/treatment cycles) (12, 13). The simulation time frame is set to 10 years.

**Table 1:** Transition probabilities.

	**Baseline value**	**Lower limit**	**Higher limit**
**BEV**+**LOM**			
P_PFS − PFS − 1_	0.77	0.62	1.00
P_PFS − PD − 1_	0.15	0.12	0.18
P_PFS − death − 1_	0.07	0.06	0.09
P_PD − PD − 1_	0.87	0.69	1.00
P_PD − death − 1_	0.13	0.11	0.16
**LOM**			
P_PFS − PFS − 2_	0.55	0.44	0.66
P_PFS − PD − 2_	0.37	0.30	0.44
P_PFS − death − 2_	0.08	0.06	0.09
P_PD − PD − 2_	0.91	0.73	1.00
P_PD − death − 2_	0.09	0.07	0.11

### Model parameters

From the perspective of the Chinese healthcare system, cost predictions were conducted (Table 2). In the analysis process, all medication costs, diagnostic fees (such as thyroid function and metabolic testing), treatment expenses for disease progression, management fees for grade 3, 4 adverse events, and hospitalization charges were taken into account. Implicit costs were disregarded due to factors like individual variability. Based on trial data by Stupp et al. (14), we computed the second-line treatment expenses for the two patient groups. All costs were converted based on the January 2024 exchange rate ($1 = ¥7.16). Quality-adjusted life years (QALYs) were utilized to measure survival duration. Since the original literature did not report utility value data, we referenced previously published studies (15) and set the utility values for disease progression, death, and event-free survival as 0.89, 0.74, and 0.00, respectively. Table 1 displays the cost and benefit parameters of the model. Following the recommendations of the Chinese Pharmacoeconomic Evaluation Guidelines (16), costs and utility values were discounted at an annual rate of 5%, and sensitivity analyses were conducted.

**Table 2:** Parameters for the base case cost-effectiveness model.

**Parameters**	**BEV+LOM**	**LOM**
**Clinical efficacy, months**		
Median PFS		
	4.2	1.5
Median OS		
	9.1	8.6
**Unit price in the model, $**		
Lomustine	3.25	3.25
Bevacizumab	209.50	209.50
MRI (head)	150.84	150.84
Health questionnaire/survey	2.79	2.79
Neurocognitive testing	98.60	98.60
Electrocardiogram (ECG or EKG)	6.01	6.01
Complete blood count (CBC)	2.09	2.09
Blood biochemistry	15.92	15.92
Urinalysis	4.47	4.47
Temozolomide	67.96	67.96
CT (head)	141.06	141.06
Urokinase	20.95	20.95
Metoprolol extended-release tablet	2.05	2.05
Nitropuna	1.27	1.27
**Drug toxic effects costs per cycle, $**		
Adverse reaction management costs	3.53	0.03
**Tests costs per cycle, $**		
Medical examination fee	464.67	459.04
**Disease status utility per year, QALY**		
Utility of PFS	0.89	0.89
Utility of PD	0.74	0.74
**Discount rate, %**	**5**	**5**

### Statistical analysis

ICER stands for Incremental Cost-Effectiveness Ratio, which represents the ratio of the change in cost (in US dollars) to the change in effectiveness (measured in quality-adjusted life years) between two treatment options, used to calculate cost-effectiveness. If the ICER is below $35,906 per QALY, it is considered cost-effective, representing three times the per capita GDP of China in 2022 ($35,906 per QALY).^1^ Sensitivity analysis is performed on each model variable to determine which variables have the greatest impact on the cost-effectiveness ratio. We conducted probability sensitivity analysis using Monte Carlo simulation with 1,000 iterations, allowing us to simultaneously consider uncertainties in costs, health utilities, and other aspects to better assess the uncertainty of the model.

## Results

### Base-case analysis

**Figure 2:**
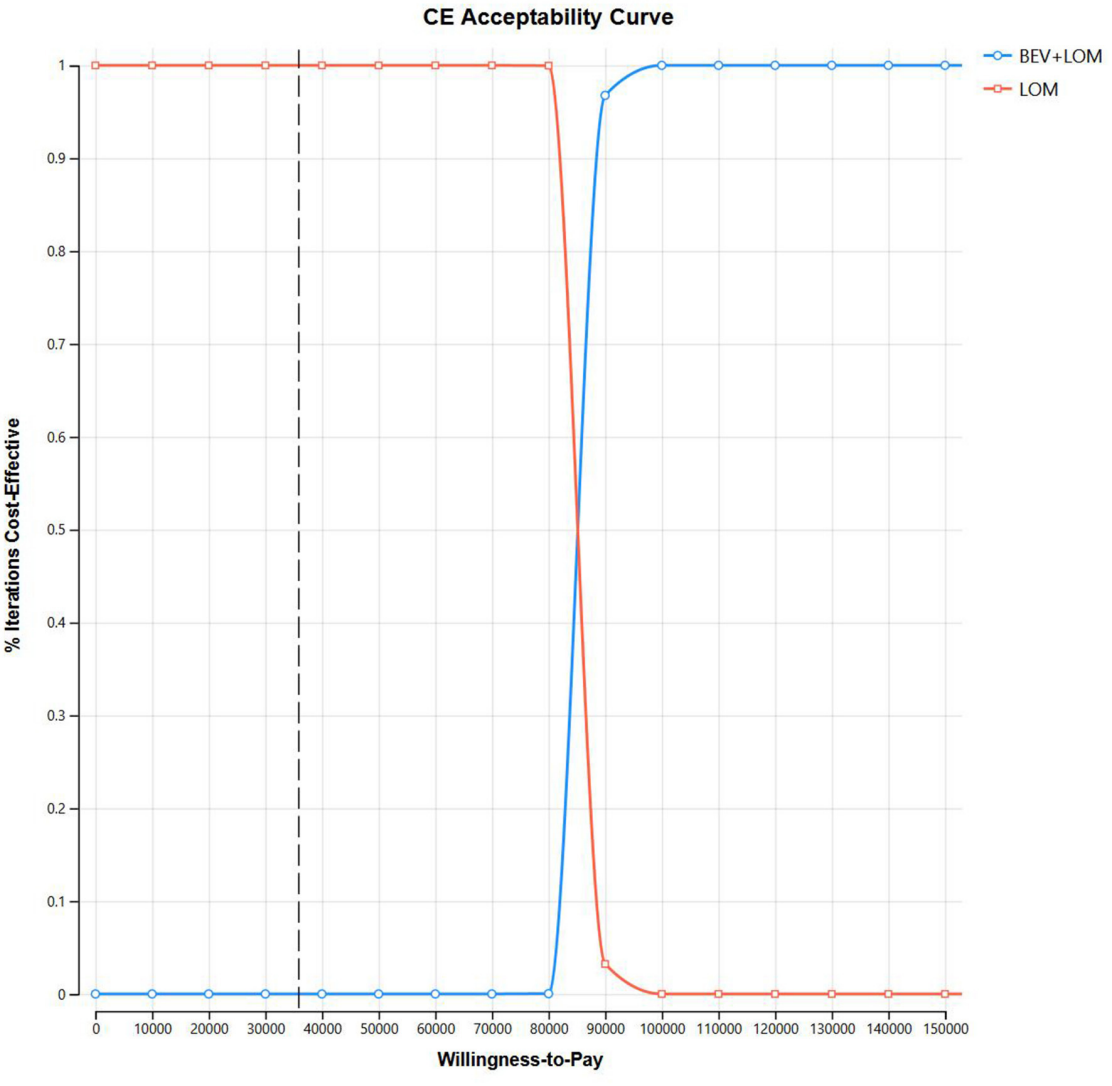
Cost-effectiveness acceptability curves.

Our foundational analysis results indicate that when comparing the combination therapy of BEV+LOM with LOM monotherapy, the total treatment cost increased from $2,646.70 to $23,650.98. The health-adjusted life years (QALYs) for the BEV+LOM combination therapy rose from 0.26 QALYs to 0.51 QALYs, an increase of 0.25 QALYs. Therefore, the incremental cost-effectiveness ratio (ICER) stands at $84,071.12 (Table 3). The cost-effectiveness curve illustrates that within the willingness-to-pay (WTP) range ($35,906/QALY), BEV+LOM combination therapy is not a cost-effective treatment option for patients with unresectable malignant pleural mesothelioma, unless there are suitable funding or drug donation programs and health insurance policy coverage (Figure 2).

**Table 3:** Results of the cost-effectiveness analysis.

**Parameters**	**LOM+BEV**	**LOM**
Costs of PFS	22,060.65	1,206.50
Costs of PD	1,590.33	1,440.20
Utility of PFS	0.44	0.19
Utility of PFS	0.07	0.07
Total costs	23,650.98	2,646.70
Total effectiveness	0.51	0.26
Incremental costs	21,004.28	/
Incremental effectiveness	0.25	/
Total C/E	46,374.47	10,179.62
ICER $/QALY	84,071.12	/

### Sensitivity analysis

**Figure 3:**
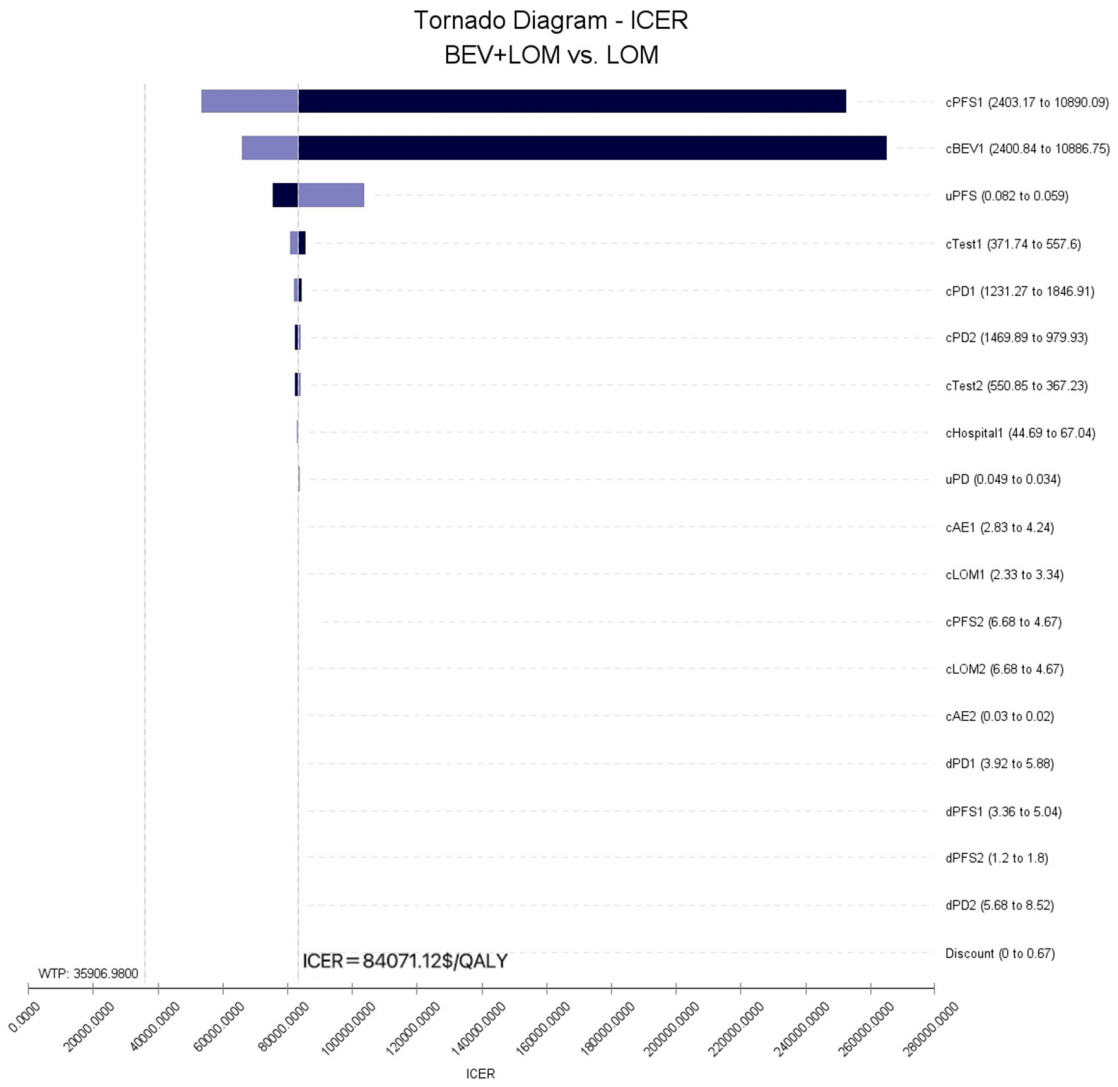
Tornado diagram of one-way sensitivity analysis.

**Figure 4:**
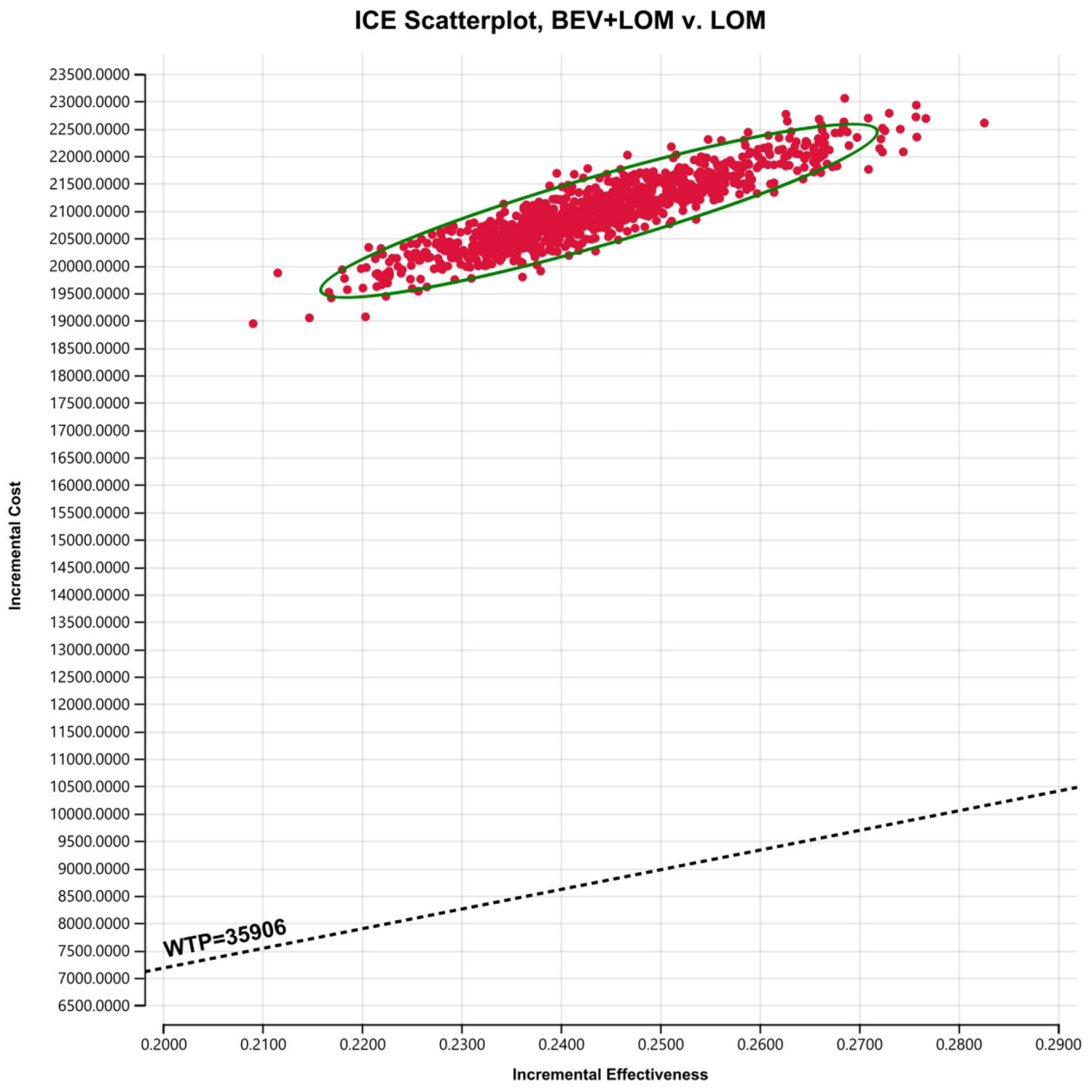
Scatter plot for probabilistic sensitivity analysis.

To assess the impact of the Markov model parameters created in this study on the research results, we conducted univariate sensitivity analysis. The results were depicted using tornado diagrams (Figure 3). The parameters with the greatest influence in the model were the costs of the PFS state in the BEV+LOM group, the drug costs of bevacizumab, and the utility value of the PFS state. In univariate sensitivity analysis, changes in the costs associated with managing grade 3, 4 adverse events, the testing methods used, or hospital expenses had the least impact on the predicted incremental cost-effectiveness ratio (ICER). Variations in all parameters did not lead to significant changes in the results, indicating the robustness of the model. The results of probabilistic sensitivity analysis (1,000 iterations) consistently showed that the ICER remained above $35,906/QALY (Figure 4).

## Discussion

Markov model is a mathematical model used to describe stochastic processes, which can be used to describe the state changes of complex systems, simplify the process of clinical disease occurrence and development, and be used for rapid evaluation of health technology.

Bevacizumab is a monoclonal antibody that targets vascular endothelial growth factor (VEGF) in the bloodstream. It has been approved in the United States for the treatment of recurrent high-grade gliomas but has not received approval in Europe. In the largest randomized trial to date for progressive glioblastoma, 437 patients experiencing their first glioblastoma progression after radiotherapy and temozolomide were randomly assigned to receive either bevacizumab plus lomustine or lomustine alone. Compared to lomustine alone, the combination of lomustine and bevacizumab improved the objective response rate (41.5% vs. 13.9%) and progression-free survival (4.2 months vs. 1.5 months) but did not improve overall survival (9.1 months vs. 8.6 months; HR 0.95, 95% CI 0.74–1.21). Bevacizumab can also cause various toxicities, including cardiovascular effects (such as hypertension, thromboembolism, and left ventricular dysfunction) and non-cardiovascular effects (such as proteinuria, delayed wound healing, and bleeding). In terms of economic value, compared to single-agent LOM therapy, the total treatment cost increased from $2,646.70 to $23,650.98 with BEV+LOM combination therapy. The health-adjusted life years (QALYs) with BEV+LOM combination therapy increased from 0.26 QALYs to 0.51 QALYs, resulting in an increase of 0.25 QALYs. Therefore, the incremental cost-effectiveness ratio (ICER) was $84,071.12. The cost-effectiveness curve indicates that within the willingness-to-pay (WTP) range of $35,906 per QALY, BEV+LOM combination therapy is not a cost-effective treatment choice for unresectable malignant pleural mesothelioma patients unless there is suitable funding or drug donation programs and health insurance coverage.

From the perspective of the Chinese healthcare system, cost predictions were made. In the analysis process, all medication expenses, diagnostic costs (such as thyroid function and metabolic testing), treatment expenses for disease progression, management costs for grade 3, 4 adverse events, and hospitalization costs were considered, including the expenses for second-line treatment. Currently, there exists a significant debate within the medical community regarding the appropriate determination of hospital payment thresholds. Specifically, in the context of the United States healthcare system, the delineation of willingness-to-pay (WTP) thresholds for anticancer drugs remains a topic of contention. Typically, this threshold is estimated to range between $100,000 and $150,000 per quality-adjusted life year (QALY). In contrast, for non-cancer drugs, the established range for WTP thresholds is generally acknowledged to span from $5,000 to $100,000 per QALY (17). Despite an ICER exceeding this threshold, many anticancer drugs, especially new biologics, are still widely used. In this study, the WTP was set at three times the per capita GDP according to WHO standards, but whether this WTP is fair for some rare diseases remains unclear. In economic evaluations of different fields and diseases, comprehensive treatment approaches currently yield different results. In a study on first-line treatment for metastatic renal cell carcinoma, it was found that the use of nivolumab with ipilimunab could increase QALYs by 0.96, with a cost of $108,363 per QALY. Therefore, although the cost-effectiveness curve of this study shows that within a willingness-to-pay (WTP) range of $35,906 per QALY, BEV+LOM combination therapy is not a cost-effective treatment choice for patients with unresectable malignant pleural mesothelioma, whether different regions in China need to adjust the WTP appropriately based on the severity and rarity of different interventions and diseases is still debatable.

Our study developed a Markov decision tree model to simulate the disease process. However, there are still the following limitations: the cost-benefit analysis model is based on second stage clinical trials rather than real-world population data, and there are no Chinese patients involved. Future research can further explore from the following perspectives. In addition, the medical expenses of this study are derived from drug price disclosure websites and local hospitals, thus, the research results have timeliness.

In summary, the results of this study indicate that, from the perspective of Chinese taxpayers, the first-line regimen of BEV in combination therapy is not economically feasible for patients with progressive glioblastoma under the current medical insurance policies and environment. However, in order to provide more affordable treatment for this rare patient population and improve their quality of life, promoting appropriate drug donation programs and social assistance should be encouraged.

## Data availability statement

The raw data supporting the conclusions of this article will be made available by the authors, without undue reservation.

## Author contributions

ZC: Data curation, Methodology, Writing – original draft, Writing – review & editing. FT: Conceptualization, Data curation, Writing – original draft. YZ: Data curation, Formal analysis, Writing – original draft, Writing – review & editing.

## Footnotes

^1^Available online at: https://www.stats.gov.cn/sj/zxfb/202302/t20230228_1919011.html (assessed January 2024).
